# Aneurism—Cases from Practice

**Published:** 1891-10

**Authors:** Edmund Carleton


### ANEURISM.—CASES FROM PRACTICE.

PROF. EDMUND CARLETON, M. D.

Read before the International Hahnemannian Association, Richfield Springs, June, 1891.

The subject of aneurism has been one of peculiar interest to our profession for a long time. It has presented difficulties not easy to be overcome. The medical and surgical wings of the old school have devoted their best thoughts and experiments to the cause. The former has accomplished a little in the line of contraria; Opium has retarded the blood current and facilitated local stasis in a very few instances; Gallic acid and Iron have thickened the blood, and in that way helped to the formation of a clot in the sac, successfully in enough cases to over-balance the harm done in others; and that is about all.

I well recollect a case of aneurism of the arch of the aorta, in 1873 or thereabouts, which attracted considerable attention at the time. The subject of it was a middle-aged gentleman, who had been active in mercantile life, while living for a number of years in a tropical climate. He returned to this country in a disabled condition. His physician advised with a well-known professor of surgery, and together they decided to try the Iron and acid treatment. The patient was kept on his back, had little food or drink, and swallowed enormous doses of medicine. When the constitutional symptoms resulting therefrom became alarming, the doses were reduced, to be again brought to a maximum as soon as audacious prudence permitted. After a number of weeks had elapsed, the local trouble abated; and finally a recovery was announced, with great publicity and display. The poor fellow was nearly used up by the treatment, however, and never rallied from it. I doubt if any considerable number of the physicians and students who heard of the case and its recovery ever became aware of the fact that the man perished very soon after from congestion of the lungs. To my mind, drugs were responsible for the congestion.

I have not presented this history to find fault with it, but merely to show how inadequte is the contrary method of prescribing. Other cases of cure with Iron and acid have been reported. There are no means at hand of verifying them, but presumably recovery was permanent. Due credit should be given for every real cure of such a formidable disease.

The surgical wing of the profession presents a better record. The Hunterian method of ligation has often proved successful. Inapplicable, of course, to such a case as the one just alluded to, it nevertheless stands as a monument to its illustrious originator, and as one of the legitimate means for selection by the practicer of the art of healing.

Whether knowledge of more drugs will in future render ligation superfluous is a matter of speculation. Compression, in its various modifications, has worked well sometimes. From the forcible flexion of an extremity to the digital compression of the femoral artery by a relay of students, though sometimes accompanied with dramatic incidents and perhaps newspaper notoriety, may be found the range of a mechanical process which has stood the crucial test of experience. Acupuncture, and the chemical and electrical methods, have not shown so good results. The latest plan is to fortify the weak wall of the blood-vessel by the formation of a white thrombus, starting at the inner surface of the sac. In an address on aneurism, delivered before the Midland Medical Society last year, by William Macewen, M. D., of Glasgow, the physiology of the formation is set forth in detail. The main thing used is a delicate steel pin, which is also strong, and long enough to pass through the blood-vessel and irritate its opposite wall. The blood current, as it pulsates, causes the point of the pin to scratch the inner coat of the artery. The scheme being the result of mature thought and of experiment by an able and experienced surgeon, is worthy of attention. He reports four cases treated by his method, with flattering results.

But the well-balanced homœopath is enabled to prescribe the best medicine for the individual, according to the law of cure, and to supplement it with such operative measures as the case may require. Who in this assembly can doubt the superiority of the new method of practice over the other? The following cases are cited to illustrate my views:

Ten years ago, Mrs. D., aged fifty, complained of a throbbing, choking sensation at the base of the neck and behind the sternum, worse when exerting muscular strength or when excited. The examining finger in the supra sternal fossa clearly defined the lesion. It was an aneurism of the arch of the aorta. Later on it became so large that a person sitting upon the opposite side of the room could easily discover a bulging, palpitating part just above the sternum. At that time she was much troubled with dizziness, pain in temples, timidity, sighing, and sadness. Those who wish to account for symptoms, will be interested to hear that she naturally would have been sorrowful, by reason of repeated family afflictions. Her husband and all but one of her numerous children had died in quick succession. She never was informed of the nature of her malady, but advised not to make any great exertion. The symptoms called for Ignatia, and that was the remedy she received, in the 200th potency. The frequency of its administration depended upon the violence of the symptoms; when very bad, she took a teaspoonful of watery solution every few hours; at other times, only morning and night, or omitted a number of days together.

The aneurism continued to grow slowly for months after I began to give Ignatia. I had no expectation of curing that, but did hope to make the patient tolerably comfortable. She felt and acted better, ate and slept more, and improved in flesh. Some months later, it became evident that the tumor had ceased to grow, and then it slowly and steadily diminished in size. Strength returned. She took up the active occupation of nursing and has continued at it since. It was my desire to have you examine her at this meeting, but a patient who is particularly fond of her objected to being left alone, and the plan failed; but an interview can be arranged for those of you who are sufficiently interested to visit her. You will find the arch somewhat enlarged and solidified. This case has been shown to other physicians at different times by me. The diagnosis and recovery have not yet been questioned. Nevertheless, if this paper comes to the notice of the old-school statistician, he probably will add one more to his tally of "spontaneous recoveries." But to-day you are the jury, and I am content to abide by your verdict.

A case of popliteal aneurism came under my observation last winter at Ward's Island, during my term of attendance, Wm. B. Breck, M. D., and, later, L. E. Poole, M. D., House Surgeons. G. T. Stewart, M. D., Chief of the House Staff, kindly furnishes the history.

A. B., aged thirty-two, born in Canada, single, waiter, admitted January 5th, 1891. Good family history. Had diseases incident to childhood. Hard drinker. Has had gonorrhœa repeatedly. Eczema three years. Subject to epistaxis.

About six weeks before admission, first noticed a tumor in left popliteal space, about the size of a hickory nut. At first it did not throb. Then it began to throb intermittently; pains not constant but paroxysmal, every two or three hours. Tumor increased very rapidly in size, pain became constant, night and day, shooting down to heel, so excruciating that it almost caused fainting; cold sweat accompanied it.

Physical examination disclosed a hard tumor in popliteal space, the size of a walnut, painful to pressure; heart sound heard over same; pulsation in tumor ceased and it became softer on compressing femoral artery in Scarpa's triangle. Leg œdematous; ulcer on lower third; limb semi-flexed; extension impossible; patient said that "hamstrings seemed as if grown together." Temperature, 100° to 101°.

On the 14th of January, I decided to try the method of compression devised by Dr. Walter Reid, of the British Army. This consists in applying an Esmarch bandage from the toes up to the aneurism, passing the latter without compressing it, by making a diagonal turn-up alongside the knee-joint, and then continuing the bandage far enough up the thigh to make sure of a good place for the rubber tubing, which is fastened tightly around the limb, and the bandage then removed. This method has been reported successful in numerous cases. Interest in it and the case in hand brought physicians and students to witness the trial, which was made under ether. The apparatus remained in place three hours and a half. Few of us could wait to learn the result; but Dr. Breck wrote to me that night the following particulars:

"I am sorry to inform you of the unfavorable result of the attempt to cure the aneurism this afternoon. At 7 P. M., the Chief of Staff being present, I gradually loosened the Esmarch. At first there was no pulsation, but before the last coils were removed, it came back with almost as much strength as before the application. Digital compression was immediately applied, though causing the patient extreme pain, and before anything could be done to relieve it, his pulse commenced to fail rapidly, finally becoming imperceptible at the wrist, the patient seeming to be going into collapse. Stimulants were given and he rallied nicely, but further manipulations seemed to be contra-indicated, so nothing more was done. At present, 11 P. M., the patient is resting without narcotics, though still suffering much pain, mostly at the point where the bandage was fastened."

I resolved to make further trial of compression, and this time with the aneurism tourniquet. By January 23d, the patient had rallied sufficiently to permit the operation, which was then performed, at Scarpa's triangle, with the large tourniquet. This is Dr. Breck's report:—"At 11.30 A. M., applied the tourniquet. Comparatively little pressure caused the pulsation in the tumor to almost cease, and we left the patient quiet and comfortable with the instrument in place. About two hours afterward, was called to see him, and found him nearly frantic with pain. Measures were taken to relieve him, but he insisted on the removal of the tourniquet, saying he would prefer to have his leg off and be done with it at once. This is now the second unsuccessful attempt, and the tumor seems to be gradually growing in size."

Another unsuccessful attempt was made on January 26th, causing dangerous symptoms. Mr. B. had now become resigned to any plan which promised relief, even amputation. The swelling seemed as large as an infant's head. Every heart's-beat caused a pulsation in the limb, which could be seen easily across the room. After due consideration, it was decided to perform the Hunterian operation next, and to apply the ligature to the femoral artery in the middle of the thigh, thinking thus to offer a safer retreat in case of disaster, necessitating amputation, than if the ligature were in Scarpa's space.

This plan was carried into execution, in the presence of physicians and students, January 29th, with the aid of ether and the Esmarch apparatus. Our antiseptic friends would probably not approve of the course that was followed, as we relied upon simple cleanliness, as usual, all through the operation, it being in a large, full hospital. The artery was found in the sheath with the vein, and *in front of the vein instead of behind it.* This anomaly is unique so far as I can learn. Well-waxed, braided silk, No. 5 size, was tied tightly around the artery, and one end left hanging outside the wound, the other cut short. Were the operation to be repeated by me to-day, both ends would be cut short. The wound was carefully rinsed with dilute Calendula, then dried, the sides approximated with ordinary, interrupted sutures, and dry cotton (unmedicated) bound over the incision. The hospital record of what followed reads thus: "Patient rallied, but in the evening, about seven o'clock, he suffered excruciating pain. Doctor gave him seven-eighths of a grain of Morphine, and other drugs, but the pain kept increasing. At 11 P. M., he could stand the pain no longer, and upon consultation of staff, an amputation was deemed necessary. So Drs. Breck and Miller went to city for Dr. Carleton's consent to operate. But Dr. Carleton, after getting the patient's symptoms from the doctors, decided to prescribe instead of amputate, and said if pain did not cease he would amputate in the morning. He sent Coffea$^{200}$, a few pellets to be put upon the tongue, every fifteen minutes, until pain should abate, and then stop. After receiving two doses of medicine, the pain abated, and patient slept soundly. When he awoke the pain was nearly all gone, and he was feeling well in all respects. Dr. Carleton was notified in the morning of good recovery and he did not deem it necessay to come over. The temperature at 11 P. M., when the doctors went to the city, was 104°; at 4 A. M., January 30th, it was 102°; at 8 A. M., 101.3°. January 31st, temperature was 101.3° in A. M.; 102° in P. M.

Patient doing nicely. February 1st, A. M., 100.3° ; P. M., 101°. February 2d, A. M., 101° ; P. M., 99°. February 3d, A. M., 99° ; P. M., 101.4.°

"Bowels were constipated and patient was very restless all day, but was quiet at night and slept most of the night.

| | | | | | |
|---|---|---|---|---|---|
| February | 4, | A. M., | 99° ; | P. M., | 100°. |
| " | 5, | " | 100° ; | " | 100.3°. |
| " | 6, | " | 100.2° ; | " | 100.4°. |
| " | 7, | " | 99° ; | " | 99.2°. |
| " | 8, | " | 99.2° ; | " | 99.3°. |
| " | 9, | " | 99.2° ; | " | 99.4°. |

"Temperature ranged from 99.4° down to normal and stayed there. Wound healed by granulation. Very little pain at times—recovery was all that could be looked for."

The hospital narrative may be amplified a little. The seven-eighths of a grain of Morphine had been followed by a huge dose of bromides, and that by a large dose of Chloral, and that by three ounces of whiskey. None of these made any apparent impression upon the case. The patient screamed and tossed, and wanted to throw himself out of the window. The symptoms that led me to select Coffea were "pains seemed insupportable, driving to despair;" "great nervous agitation and restlessness." These tally exactly with Hering's *Materia Medica*. Besides, patient complained of "arterial tension," twisting and wrenching, where the ligature had been applied, and running thence up to the heart and brain, which corresponds pretty fairly with Hering's symptom, "strong, quick palpitation of heart with extreme nervousness, sleeplessness, and cerebral erethism." It is my present belief that Coffea was his remedy from the start. Do not understand me as expressing the opinion that Coffea would have cured the aneurism ; nor that it would not ; but it would have done good if given sooner than it was. The great fact to which your attention is called is that the similar remedy will produce euthanasia better than the contrary can. We all know that it will *cure* better.

The stitches came away with a little pus. The ligature came away March 11th, the fortieth day after its application. Artificial heat was applied to the entire extremity, immediately after the operation, of course, and, as a matter of precaution, maintained a number of days; but the leg never became cold nor pale, showing that the collateral circulation had become somewhat established before the artery was tied. Pulsation has not yet been detected anywhere below the ligature. The ulcer healed gradually. There was, for a number of days, occasional pain in calf of leg and toes—non-characteristic and not very distressing. The limb was numb, weak, and clumsy for some time. Patient cannot yet make a complete extension, and walks mostly upon the toes and ball of foot. The tumor steadily decreased in size. It can yet be felt in the popliteal space, round, hard, and tough. Although a few of the objective symptoms remain in slight degree, yet they are nearly gone and are going. Practically the man is cured. He left the hospital the second week in May.

